# Comparing the gut microbiota of Sichuan golden monkeys across multiple captive and wild settings: roles of anthropogenic activities and host factors

**DOI:** 10.1186/s12864-024-10041-7

**Published:** 2024-02-06

**Authors:** Xuanzhen Liu, Jianqiu Yu, Zongjin Huan, Mei Xu, Ting Song, Ruilin Yang, Wei Zhu, Jianping Jiang

**Affiliations:** 1 Chengdu Zoo & Chengdu Research Institute of Wildlife, 610081 Chengdu, China; 2 Chengdu Institute of Biology, Chinese Academy of Sciences, 610041 Chengdu, China

**Keywords:** Antibiotics, Captivity, Firmicutes/Bacteroidetes, Microbiome, Non-human primate, Pedigree

## Abstract

**Background:**

Captivity and artificial food provision are common conservation strategies for the endangered golden snub-nosed monkey (*Rhinopithecus roxellana*). Anthropogenic activities have been reported to impact the fitness of *R. roxellana* by altering their gut microbiota, a crucial indicator of animal health. Nevertheless, the degree of divergence in gut microbiota between different anthropogenically-disturbed (AD) *R. roxellana* and their counterparts in the wild has yet to be elucidated. Here, we conducted a comparative analysis of the gut microbiota across nine populations of *R. roxellana* spanning China, which included seven captive populations, one wild population, and another wild population subject to artificial food provision.

**Results:**

Both captivity and food provision significantly altered the gut microbiota. AD populations exhibited common variations, such as increased Bacteroidetes and decreased Firmicutes (e.g., *Ruminococcus*), Actinobacteria (e.g., *Parvibacter*), Verrucomicrobia (e.g., *Akkermansia*), and Tenericutes. Additionally, a reduced Firmicutes/Bacteroidetes ratiosuggested diminished capacity for complex carbohydrate degradation in captive individuals. The results of microbial functional prediction suggested that AD populations displayed heightened microbial genes linked to vitamin and amino acid metabolism, alongside decreased genes associated antibiotics biosynthesis (e.g., penicillin, cephalosporin, macrolides, and clavulanic acid) and secondary metabolite degradation (e.g., naphthalene and atrazine). These microbial alterations implied potential disparities in the health status between AD and wild individuals. AD populations exhibited varying degrees of microbial changes compared to the wild group, implying that the extent of these variations might serve as a metric for assessing the health status of AD populations. Furthermore, utilizing the individual information of captive individuals, we identified associations between variations in the gut microbiota of *R. roxellana* and host age, as well as pedigree. Older individuals exhibited higher microbial diversity, while a closer genetic relatedness reflected a more similar gut microbiota.

**Conclusions:**

Our aim was to assess how anthropogenic activities and host factors influence the gut microbiota of *R. roxellana*. Anthropogenic activities led to consistent changes in gut microbial diversity and function, while host age and genetic relatedness contributed to interindividual variations in the gut microbiota. These findings may contribute to the establishment of health assessment standards and the optimization of breeding conditions for captive *R. roxellana* populations.

**Supplementary Information:**

The online version contains supplementary material available at 10.1186/s12864-024-10041-7.

## Background

The golden snub-nosed monkey (*Rhinopithecus roxellana*) is endemic to the temperate forests of the mountainous highlands (1,500–3,400 m above sea level) in central and southwestern China. Their diet includes buds, flowers, leaves, bark, and lichen [1]. Classified as Endangered on the International Union for Conservation of Nature Red List [2], this species holds the status of a first-class national species in China, underscoring its significance in conservation efforts. Captive breeding stands as a crucial strategy for the preservation of this valuable species, with over 50 organizations housing captive *R. roxellana* by 2019 [3]. Records from 1955 to 2016 indicate 898 captive individuals, of which 673 were born in captivity [4]. Despite dedicated efforts to maintain and improve the health and fitness of captive individuals in recent decades, they remain susceptible to gastrointestinal diseases, such as diarrhea and dyspepsia, compared to their wild counterparts [5–7]. This susceptibility may be attributed to dietary and environmental changes associated with captivity. Additionally, certain wild *R. roxellana* populations have been subjected to artificial food provision as a conservation measure. Although less intrusive, this anthropogenic disturbance may also exert adverse effects on the health of wild individuals [8, 9]. Therefore, elucidating the physiological changes induced by these anthropogenic activities, such as captivity and food provision, is essential for the effective conservation and breeding of this precious animal.

The gut microbiome plays a pivotal role in the health, nutrition, and physiology of wildlife [10, 11], including various endangered animals both in the wild and in captivity [12, 13]. Captivity has been documented to induce significant alterations in the gut microbiota of non-human primates, such as chimpanzees, gorillas, red-shanked doucs, and Japanese macaques [14–16]. Some of these microbial changes are associated with an elevated risk of gastrointestinal diseases in captive non-human primates [17–19]. This highlights the potential of gut microbial diversity and composition as crucial physiological indicators for assessing the health status of non-human primates [20]. *R. roxellana* belongs to colobine monkeys. This group of animals have fermenting forestomaches that are crucial for digestion and nutrition [21], underscoring the importance of studying the variations and susceptibility in their gut microbiota [22]. Recent attention has been devoted to exploring the gut microbial community structure and functions of *R. roxellana* [23–28]. Previous studies have shown that captivity significantly alters the gut microbiota of *R. roxellana* [23], with captive *individuals* exhibiting lower microbial alpha-diversity and reduced Firmicutes/Bacteroidetes ratios [29, 30]. These changes are accompanied by increased genes related to simple carbohydrate digestion, vitamin biosynthesis, and amino acid biosynthesis from carbohydrate intermediates, alongside decreased capacity for fatty acid production and fiber digestion [29, 30]. Moreover, artificial food provision has been found to impact the gut microbiota of wild *R. roxellana*, resulting in lower microbial alpha-diversity [8]. However, comparative studies involving multiple artificial populations are limited, leaving uncertainty about the commonality of these microbial changes in anthropogenically-disturbed (AD) individuals, encompassing both captive and provision-fed individuals. Given the crucial role of gut microbiota in host health, there is an urgent need for a comprehensive evaluation of gut microbial traits across different captive populations in China, especially in understanding the extent of deviation from their wild counterparts. This not only helps identify “at risk” populations, but also facilitates the optimization of the captive conditions.

Beyond its significance for species conservation, captive *R. roxellana* populations present an opportunity to investigate the determinants of the gut microbiota in non-human primates, given the well-documented physiological traits (e.g., age and gender) and pedigree relationships. Physiological factors, including age and gender, have been identified as contributors in shaping the gut microbiota of non-human primates, including *R. roxellana* [25, 31–33]. However, the significance of these physiological factors across multiple populations with diverse dietary and environmental conditions remains unclear. While host genetic effects on the gut microbiome are nearly universal [34–37], the influences of pedigree relationships on the gut microbiota of primates is a topic of debate. Although many primate studies found no strong evidence for kinship effects on gut microbiomes [38–40], a recent extensive study in baboons revealed that individuals inherit a significant portion of their gut communities from their ancestors [41]. Maternal relatives, whether residing in the same or different groups, exhibited more similar microbiota [42].

In this study, we conducted a comparative analysis of the gut microbiota in one purely wild population, one wild population subject to artificial food provision (anthropogenic disturbance), and seven captive populations of *R. roxellana*. Our hypotheses were as follows: (1) The gut microbiota of AD populations, while displaying variations among themselves, is expected to show shared compositional and functional changes in comparison to their wild counterparts. This convergence may result in an increased resemblance to the gut microbiota of humans, as observed in other captive non-human primates [14, 15]. (2) Host physiology and pedigree relationships are expected to exert significant impacts on the gut microbiota of captive individuals. We aim for this study to provide insights into the health status of various captive *R. roxellana* populations in China, and elucidating the drivers of gut microbiota variations across populations may offer valuable clues to optimize breeding conditions of the captive *R. roxellana*.

## Methods

### Sample collection and host information

We obtained permission from Wanglang National Nature Reserve, Chengdu Zoo & Chengdu Research Institute of Wildlife, Beijing Zoo, Beijing Wildlife Park, Nanjing Hongshan Forest Zoo, Shanghai Wild Animal Park, and Shanghai Zoo, and Hangzhou Zoo to collect the feces samples from *R. roxellana*. Fresh fecal samples were promptly collected after defecation using a sterile spoon, focusing on the inner part to prevent contamination. The samples were preserved in liquid nitrogen until DNA extraction. No other animal experiments were conducted in this study.

**Fig. 1:**
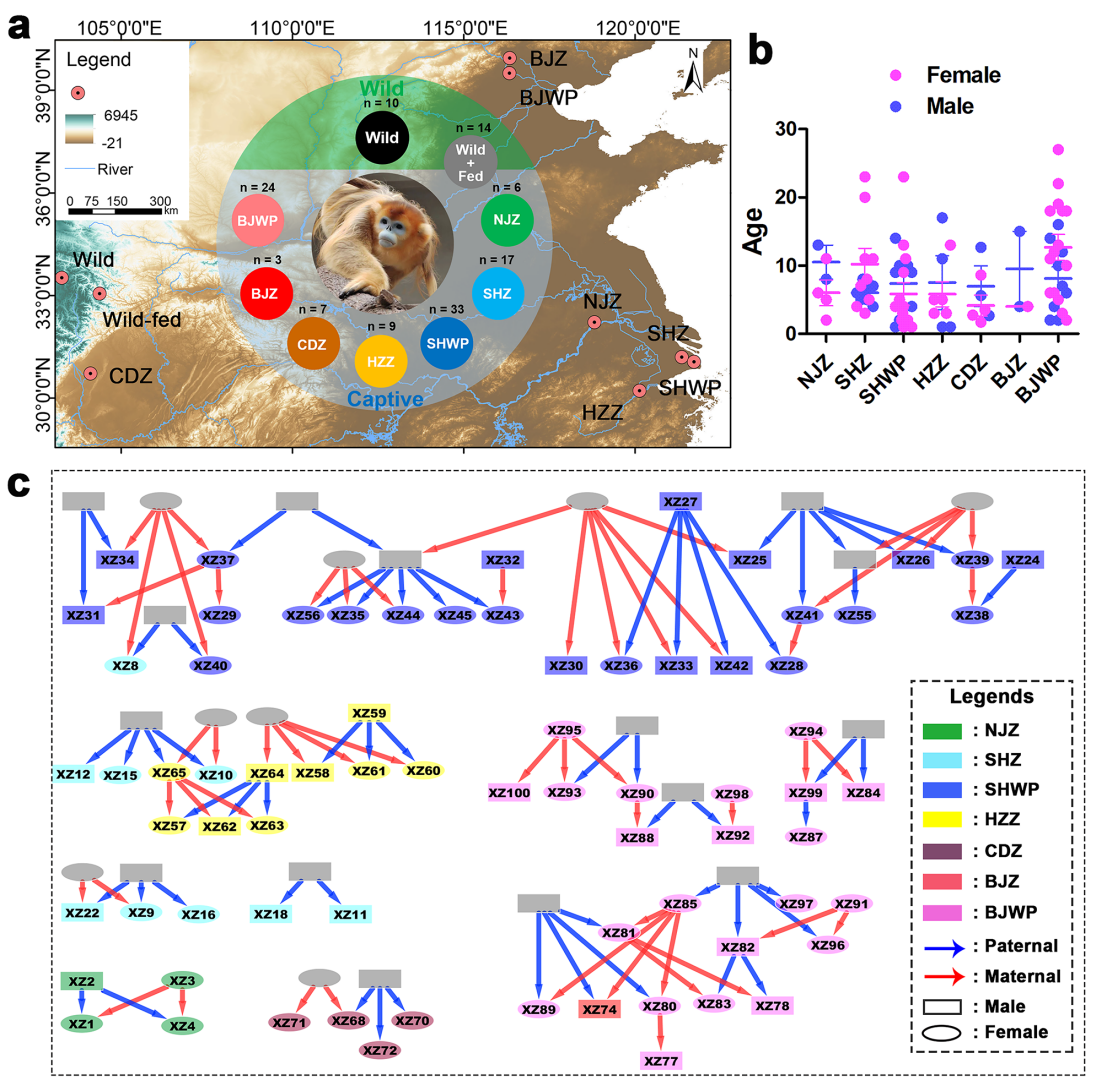
Sampling information. **(a)** Geographic distribution and sample sizes of the nine *R. roxellana* populations. The eight anthropogenically-disturbed (AD) populations included seven captive populations (NJZ, SHZ, SHWP, HZZ, CDZ, BJZ, and BJWP) and one wild population with artificial food provision (wild-fed). One wild population without anthropogenic activities (wild) is used as a control. **(b)** Age and sex structure of the hosts. No differences in ages were detected between animal sources or genders (*p* > 0.05, Scheirer–Ray–Hare test). **(c)** Pedigree relationship network of the hosts

We collected fecal samples from wild population without anthropogenic disturbance (wild, *n* = 10) in the Wanglang National Nature Reserve (103°16′E, 32°91′N) (Fig. 1a). Additionally, we collected fecal samples from a wild population with artificial food provision (wild-fed, *n* = 14) in Huangyangguan County, Mianyang City, Sichuan province, China (approximate 104°22′E, 32°63′N), near to the reserve.

For captive samples, we collected 7, 3, 24, 6, 33, 17, and 9 fecal samples from Chengdu Zoo & Chengdu Research Institute of Wildlife (CDZ), Beijing Zoo (BJZ), Beijing Wildlife Park (BJWP), Nanjing Hongshan Forest Zoo (NJZ), Shanghai Wild Animal Park (SHWP), and Shanghai Zoo (SHZ), and Hangzhou Zoo (HZZ), respectively (Fig. 1a). In each zoo or park, keepers were responsible for feces collection and ensured individual identification, with aach individual contributing one sample. The sex and age structure of the hosts is depicted in Fig. 1b. The animal pedigree relationship is illustrated in a network (Fig. 1c), with kinship indices computed for pairs of individuals to denote their genetic relatedness. Specifically, for any two individuals, we identified all common ancestors, revealing the genetic paths connecting them. The relatedness of each genetic path was calculated following the formula (1/2)^N^, where N was the total edges in this path. The sum of the relatedness of all the paths was the kinship index of these two individuals. Similar methods were applied to calculate the maternal relatedness, but only paths consisting of maternal edges were considered. The sample collection process has been seamlessly integrated into routine animal management, causing no harm to the animals and imposing no additional stress. The captive and Wild-fed individuals were considered as AD groups. Detailed host information for the samples is provided in Table S1 (Supplementary data 1).

### DNA extraction, PCR amplification, and sequencing

We performed fecal DNA extraction with MoBio DNeasy PowerSoil DNA isolation kit (Qiagen, Germany). We checked the quality of the DNA with a NanoDrop 2000 Spectrophotometer (Thermo Scientific, United States), and amplified the V4–V5 region of bacterial 16 S rRNA gene with 515 F (5-GTGYCAGCMGCCGCGGTA-3) and 909R (5-CCCCGYCAATTCMTTTRAGT-3) primers. We constructed the PCR reaction system following the methods described previously [43]. We used blank controls in DNA extraction and PCR amplification, and observed no amplification band. We sequenced the products on an Illumina Novaseq 6000 platform. We analyzed raw reads with QIIME Pipeline1 (Version 1.7.0) [44]. In the trimming analysis, we used Usearch for chimerism check to remove low-quality sequences, flash for splicing, and trimmomatic for quality control with default parameters [45]. We defined operational taxonomic units (OTUs) as sharing > 97% sequence identity, and classified representative sequences against the SILVA132 database [46]. Then, we obtained OTU tables containing taxon information (e.g., Phylum, Class, Order, Family, and Genus). We calculated the alpha- (i.e., observed OTU, Shannon index, and PD-whole-tree index) and beta-diversity indices (i.e., weighted and unweighted UniFrac distances) with QIIME pipeline. We predicted microbial functions by Tax4Fun2 [47], based on KEGG database [48–50]. We uploaded sequencing data and relevant files to Genome Sequence Archive (https://ngdc.cncb.ac.cn/gsub/) with the accession number CRA011956 (https://ngdc.cncb.ac.cn/gsa/s/y0jKJ234). The human data were obtained from a study conducted on volunteers from George Washington University Foggy Bottom campus area [51].

### Statistical analyses

Statistical analyses were conducted using IBM SPSS v21.0 (IBM, Armonk, NY, USA) and R [52]. Graphs were generated using Graphpad prism 5, ArcGis, or ggplot2, an R package [53]. Intergroup differences in alpha-diversity were assessed using Kruskal-Wallis or Mann-Whitney U tests. Principal coordinates analyses (PCoA) were performed to visualizze the similarity in bacterial composition between samples, and PERMANOVA was employed to test potential differences in bacterial composition between animal populations. It is worth noting that the gut microbiota of the newly born individual (XZ73 from CDZ, approximate 30 days old) exhibited substantial differences compared to other samples (Fig. 1a − b) and was therefore excluded from subsequent analyses.

To elucidate the impact of anthropogenic activities on the gut microbiota of *R. roxellana*, we performed pairwise differential analyses (Mann-Whitney U test) between each AD and the wild populations. The overlap of differential taxa between comparisons was visualized using UpSetR [54]. We considered differential taxa shared by more than six pairwise comparisons as consistent differences between the wild and AD populations. Indicative microbial features of each populations were identified using indicspecies (a R package) [55]. Similar analytical approaches were applied for assessing microbial functional differences.

We examined the potential effects of host physiological traits (i.e., gender and age) on gut microbial beta-diversity using PERMANOVAs of vegan package. Given the significant differences in microbial composition between animal populations, we also conducted two-factor PERMANOVAs that considered both the animal sources and host physiological traits to assess the contributions of host physiological traits to the total variances. Since age emerged a significant factor, we conducted Spearman correlation analyses to identify bacterial taxa that varied with host ages.

To test whether hosts with closer genetic or maternal relatedness shared more similar gut microbiota, we conducted Spearman correlations to examine the potential associations between microbial similarity distances and genetic or maternal relatedness. Pairwise relationships with a genetic or maternal relatedness of 0 were excluded from the correlation analyses. This is because animals from different populations consistently have a genetic and maternal relatedness of 0, and the substantial inter-population dissimilarity in gut microbiota may lead to spurious associations between microbial composition and genetic and maternal relatedness.

## Results

### Differences in gut microbiota between *R. roxellana* populations

**Fig. 2:**
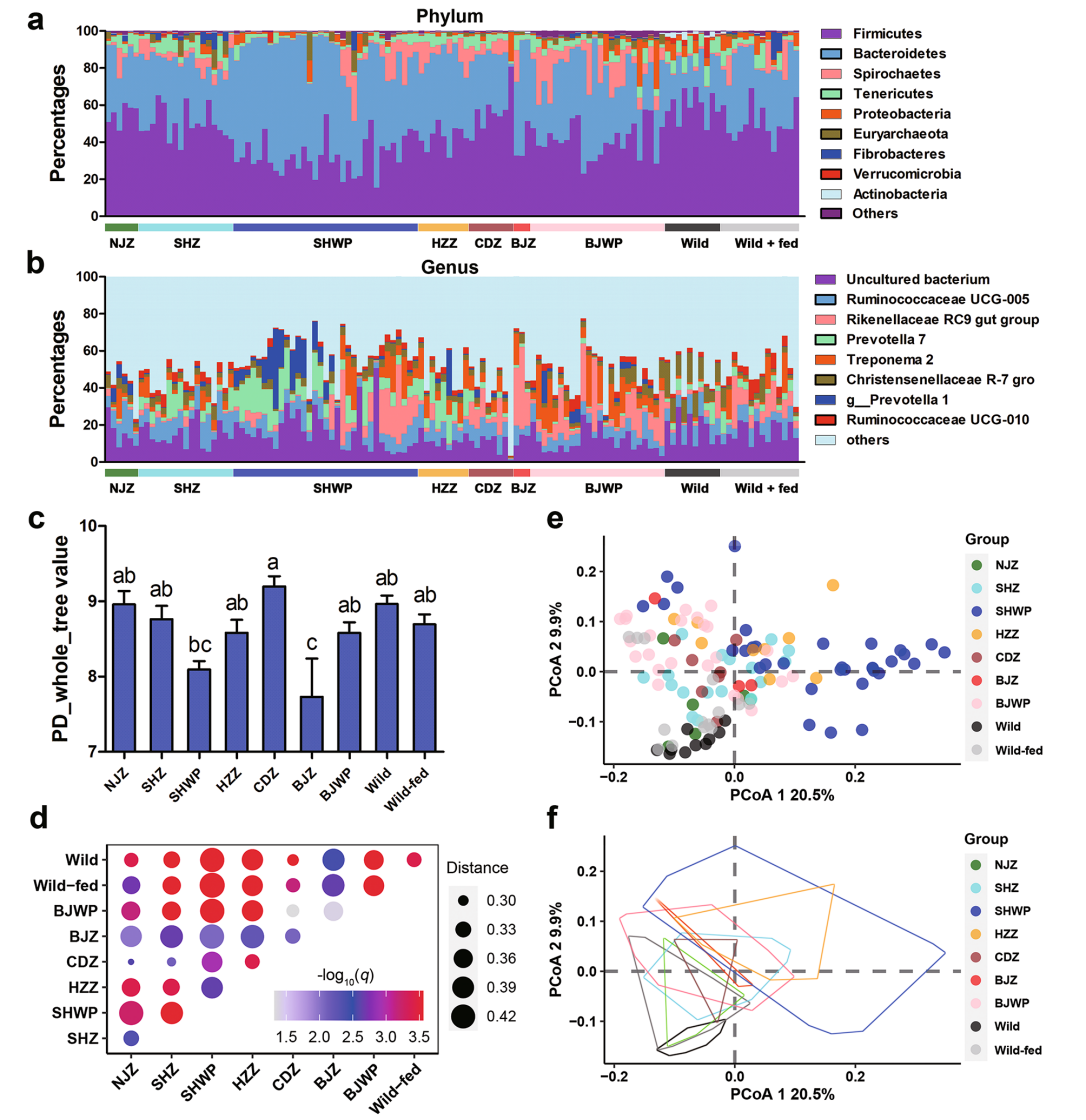
Comparison of the microbial diversity between different populations. (a − b) Bacterial composition at the phylum **(a)** and genus **(b)** levels. **(c)** Variation in microbial alpha-diversity (Shannon index) between groups. Different letters denote significant differences at a threshold of *p* < 0.05 (one-way ANOVA and S.N.K post-hoc test). **(d)** Heatmap illustrating the weighted UniFrac distances between samples from different populations. The color denotes the results of pairwise PERMANOVA on the beta-diversity (BH correction). The greater the intensity of red color, the more pronounced the statistical significance of the difference. (e − f) PCoA scatter plot **(e)** and area plot **(f)** showing the similarity in microbial composition (based on weighted UniFrac distances) between samples from different populations

The predominant gut bacteria in *R. roxellana* include Firmicutes, Bacteroidetes, and Spirochaetes at the phylum level, Ruminococcaceae UCG-005, Rikenellaceae RC9 gut group, *Prevotella 7*, *Treponema 2*, and *Prevotella 1* at the genus level (Fig. 2a − b). Among the groups, individuals from BJZ and CDZ exhibited the lowest and highest gut microbiota alpha-diversity, respectively (*p* < 0.05, one-way ANOVA and S.N.K post-hoc test; Fig. 2c). PERMANOVA results indicated significant differences in microbial beta-diversity (weighted UniFrac distances) between any two populations (*q* < 0.05, BH correction) (Fig. 2d). The PCoA scatter plot (based on weighted UniFrac distances) clearly differentiated samples from different populations, with the wild samples notably separated from the AD ones (Fig. 2e − f). The wild group exhibited the highest number of indicative bacterial taxa (e.g., Actinobacteria, Verrucomicrobia, and *Akkermansia*), followed by HZZ (e.g., Negativicutes and Selenomonadales, and *Prevotella 7*), BJZ (e.g., Rikenellaceae), NJZ (e.g., rumen bacterium NK4B4), BJWP (e.g., *Ruminobacter*), wild-fed (e.g., *Oxalobacter*) and CDZ (e.g., *Candidatus Soleaferrea massiliensis*), with the lowest in SHWP populations (Figure S1). These findings suggest that the gut microbiota in a purely wild environment differed from that in environments with anthropogenic disturbance.

### Effects of anthropogenic activities on the gut microbiota of captive *R. roxellana*

**Fig. 3:**
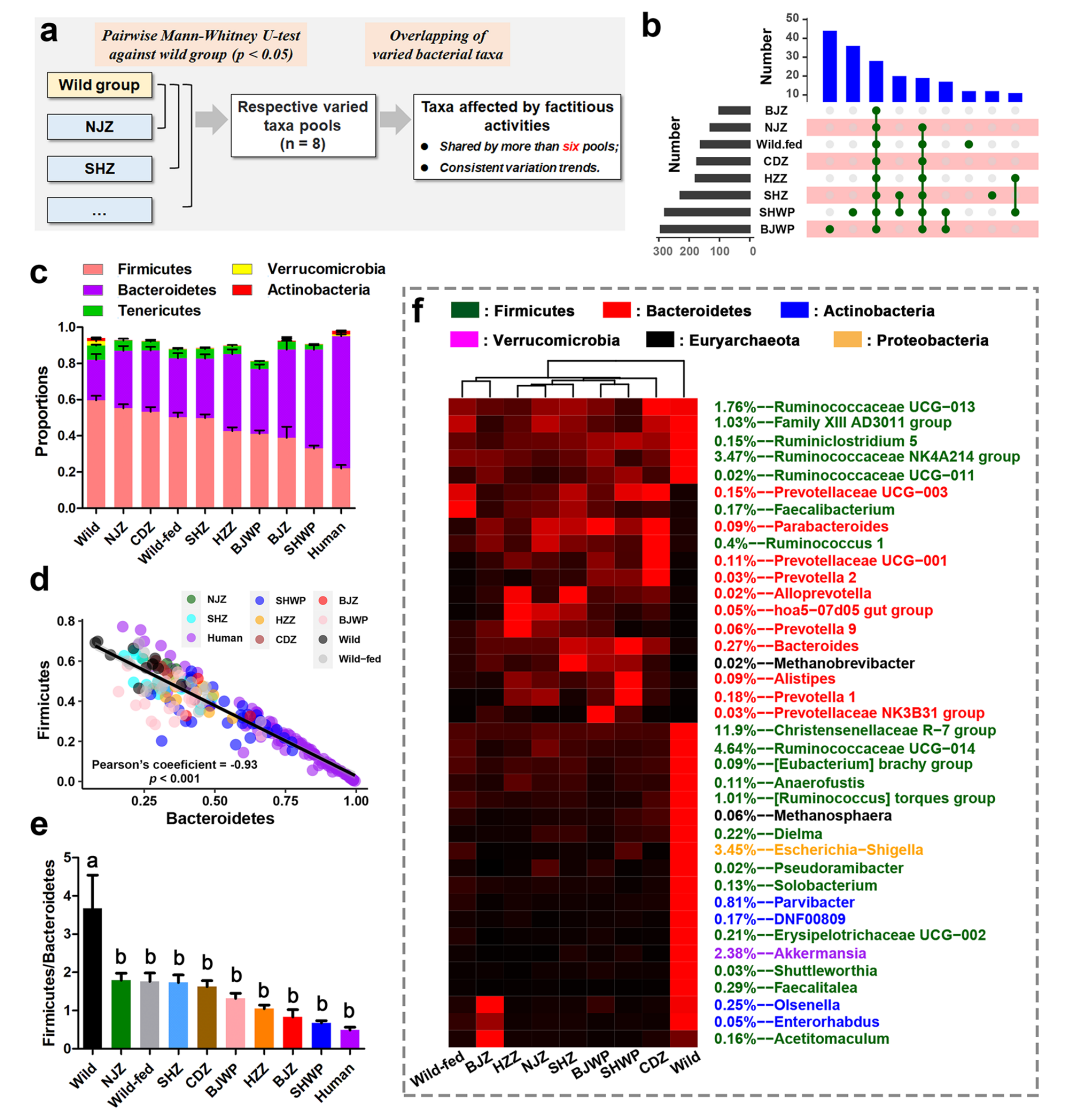
Differential analyses of gut microbial composition between captive and wild individuals. **(a)** Schematic map illustrating the workflow of differential analyses. Initial comparisons on gut microbiota were made between each AD population and the wild one population. This resulted in eight differential pools, with each differential microbe met the threshold of *p* < 0.05 (Mann-Whitney U test). Subsequently, differential microbes shared by at least six pools were considered consistently different in abundance between AD and wild individuals. **(b)** Upset plot displaying the numbers of microbes in the eight differential pools. **(c − e)** Humanized gut microbiota of captive populations at the phylum level. **(c)** Bar plot showing the proportions of the screened differential bacterial phyla. **(d)** Quantitative relationship between Firmicutes and Bacteroidetes across groups. **(e)** Ratios of Firmicutes to Bacteroidetes. Different letters denote significant difference between groups (*p* < 0.05, one-way ANOVA and S.N.K post-hoc test). **(f)** Heatmap depicting the variations in significant differential bacterial genera across groups. The average abundances of each bacterial genus were scaled to 0 − 1, where black and red colors represent 0 and 1, respectively. The colors of the row names denote the phyla

**Fig. 4:**
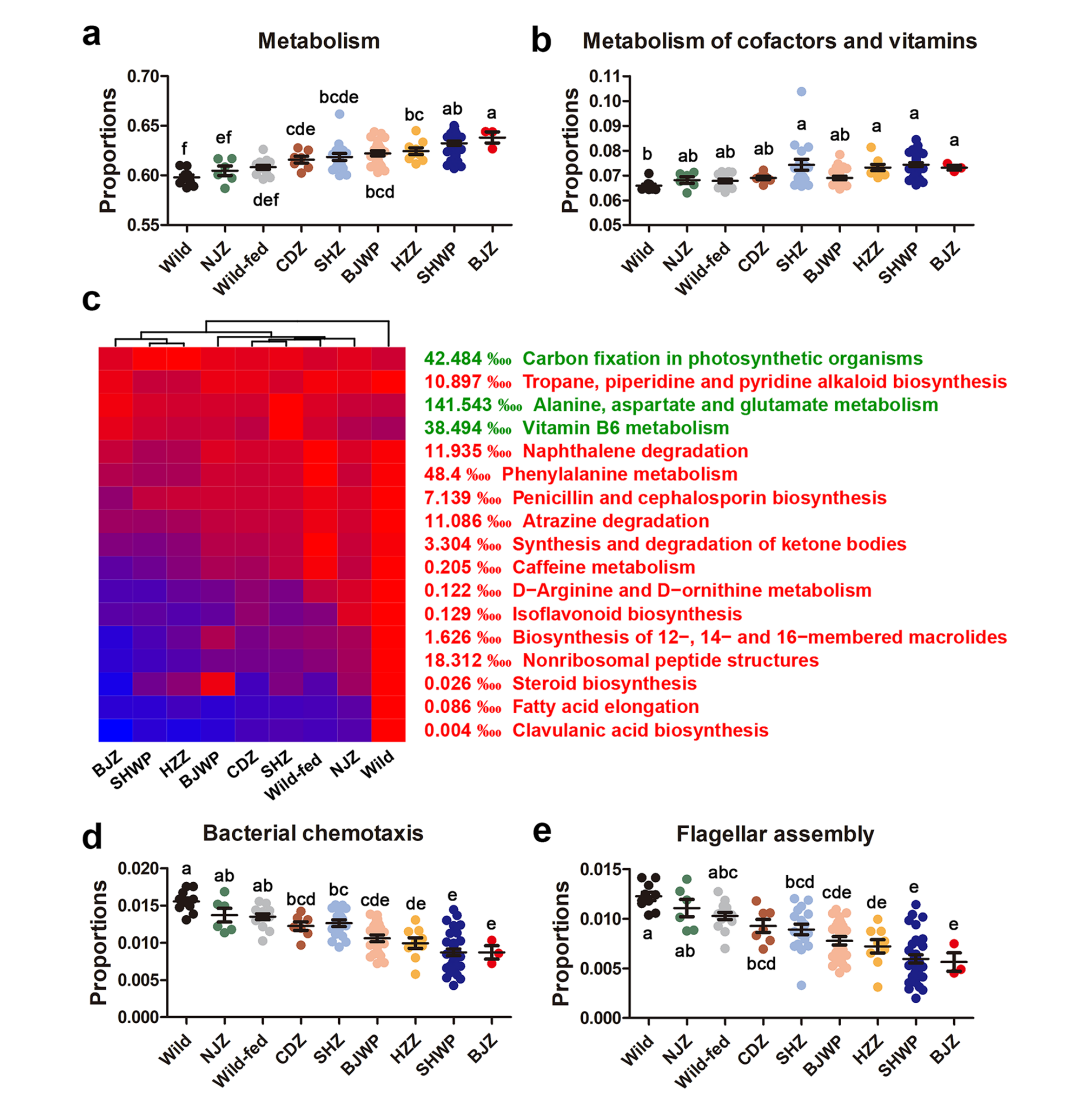
Differential analyses of gut microbial function between captive and wild individuals. The analysis flow is the same as that used for microbial compositional differences. **(a − b)** Major differential KEGG items between captive and wild gut microbiota at hierarchical levels 1 **(a)** and 2 **(b)**. Different letters denote significant difference between groups (*p* < 0.05, one-way ANOVA and S.N.K post-hoc test). **(c)** Heatmap presenting the main differential KEGG metabolic pathways at hierarchical level 3. The average abundances of each bacterial genus were scaled to 0 − 1, where black and red colors represent 0 and 1 respectively. Red and green colors of the row names denote higher and lower, respectively, in the wild gut microbiota. **(d − e)** Main differential pathways other than metabolism at level 3. Different letters denote significant difference between groups (*p* < 0.05, one-way ANOVA and S.N.K post-hoc test)

To uncover microbial changes associated with anthropogenic activities (i.e., captivity and food provision), we performed pairwise differential analyses between each AD and the wild group (Fig. 3a). Subsequently, we identified the bacteria shared by more than six differential taxa pools (*p* < 0.05, Mann-Whitney U test) as consistently affected by anthropogenic activities (Fig. 3a − b). These included five phyla, among which Firmicutes, Tenericutes, Verrucomicrobia, and Actinobacteria decreased in AD populations, while Bacteroidetes increased (Fig. 3c). A negative correlation was observed between the abundances of Firmicutes and Bacteroidetes across groups (Fig. 3d), and all AD populations, especially SHWP and BJZ groups, exhibited a reduced Firmicutes/Bacteroidetes ratio (Fig. 3e). At the genus level, AD populations displayed increased *Prevotellas*, *Bacteroids*, *Allpprevotella*, and *Alistipes*, along with decreased Christensenellaceae R-7 group, *Akkermansia*, *Parvibacter*, *Eschierichia-Shigella*, and members of Ruminococcaceae (e.g., *Ruminococcus*) (Fig. 3f and S2). Notably, an elevation in Bacteroidetes and Bacteroids abundance, coupled with a decline in Firmicutes abundance, rendered the gut microbiota of captive individuals more similar to that of humans (Fig. 3c − e and S2). Functional analyses indicated that the gut microbiota of AD individuals were enriched in genes involved in overall metabolism (KEGG level I) and cofactors and vitamins metabolism (KEGG level II) (Fig. 4a − b). At level III, AD populations showed increased vitamin B6 metabolism and alanine, aspartate and glutamate metabolism, while they lost metabolic activities in antibiotics biosynthesis (e.g., penicillin and cephalosporin, isoflavonoid, 12-, 14- and 16-membered macrolides, nonribosomal peptides, and clavulanic acid) and secondary compounds degradation (e.g., naphthalene, atrazine, and caffeine) (Fig. 4c). In addition to metabolic functions, the gut metagenomes of AD populations had fewer genes involved in cell motility (i.e., bacterial chemotaxis and flagellar assembly) (Fig. 4d − e).

### Effects of host factors on the gut microbiota of captive *R. roxellana*

**Fig. 5:**
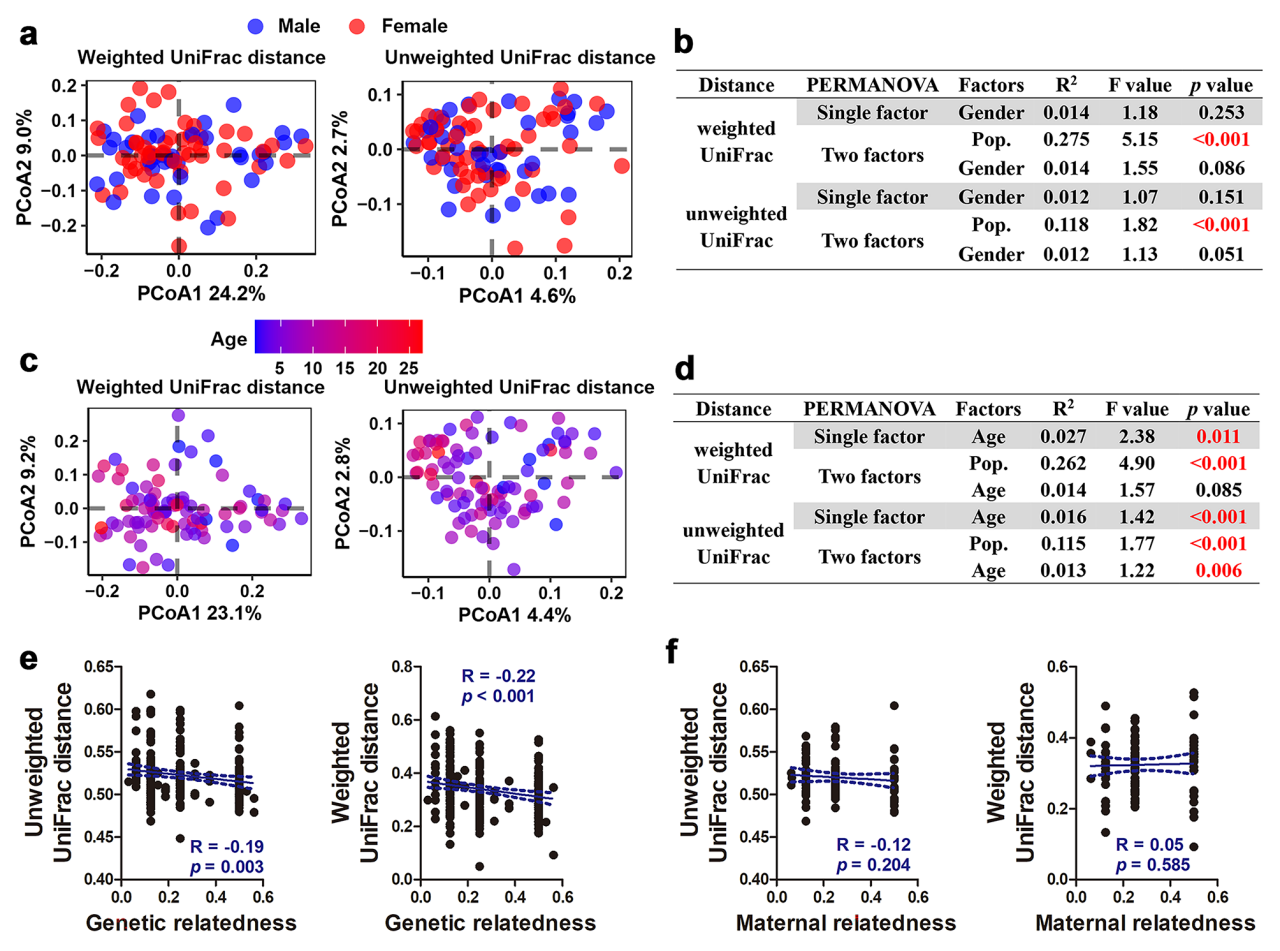
Associations of host factors (gender, age, and pedigree relationship) with gut microbiota in captive populations. **(a − b)** PCoA scatter plots showing the effects of host gender on gut microbial beta-diversity. Both the single-factor (gender) and two-factor (gender & population) PERMANOVA models were constructed to assess the significance of host gender in shaping the gut microbiota. **(c − d)** PCoA scatter plots showing the effects of host age on gut microbial beta-diversity. Both the single-factor (age) and two-factor (age & population) PERMANOVA models were constructed to evaluate the significance of host age in shaping the gut microbiota. **(e − f)** Associations of genetic **(e)** and maternal **(f)** relatedness with gut microbiota in captive populations. The associations of genetic and maternal relatedness with gut microbial similarity were analyzed using Spearman correlations. Pairwise relationships with a genetic or maternal relatedness of 0 were excluded from the correlation analyses to avoid spurious associations caused by large inter-population dissimilarity in gut microbiota

Host gender showed only marginally significant effects on the gut microbial alpha- and beta-diversity (*p* = 0.05 − 0.1 in two-way PERMANOVA; Fig. 5a − b and S3a − c), while host age was significantly associated with gut microbial diversity indices (*p* < 0.05 in both one-way and two-way PERMANOVA; Fig. 5c − d and S3d − f). The relative abundance of *Elusimicrobium*, *Oscillibacter*, OTU 822 (belonging to *Oscillibacter*), and OTU 4334 (belonging to Ruminococcaceae UCG-002) increased with host age (Figure S2g). The relative abundance of microbial genes involved in sesquiterpenoid and triterpenoid biosynthesis also increased with host age (Figure S3h). We calculated the genetic and maternal relatedness of the host and found a significant positive correlation between gut microbial similarity and host genetic relatedness (Fig. 5e), but not for maternal relatedness (Fig. 5f).

## Discussion

### Anthropogenic activities consistently impact the gut microbiota of *R. roxellana*

While the AD populations exhibiting significant differences in gut microbial composition among themselves, their distinctions from the wild *R. roxellana* populations were more pronounced (Fig. 2d − f and S1). This emphasizes anthropogenic activities as significant drivers of gut microbiota variations in this study. In line with prior studies [29, 30], we observed increased proportions of Bacteroidetes and decreased proportions of Firmicutes (e.g., *Ruminococcus*), Actinobacteria (e.g., *Parvibacter*), Verrucomicrobia (e.g., *Akkermansia*), and Tenericutes in the AD populations, resulting in a reduced Firmicutes/Bacteroidetes ratio (Fig. 3). A higher Firmicutes/Bacteroidetes ratio of the gut microbiota is linked to superiority in extracting energy from the diet [56]. Firmicutes (e.g., *Ruminococcus*), Actinobacteria (e.g., *Bifidobacterium*), and Verrucomicrobium (e.g., *Akkermansia*) phyla are associated with breaking down complex carbohydrates (e.g., cellulose and other insoluble polysaccharides) [57, 58]. Although Bacteroidetes (e.g., *Prevotella* and *Bacteroides*) also contribute to fiber utilization [59, 60], their primarily target soluble polysaccharides [61]. Studies show that coarse fiber, rather than finely ground fiber, increases the intestinal Firmicutes/Bacteroidetes ratio, reducing diarrhoea in piglets [62]. These findings suggest that captive and artificially fed *R. roxellana* populations may have a diminished capacity for coarse fiber utilization, possibly due to the loss of fiber diversity in their diet. Humanization of gut microbiota is a common occurrence in captive non-human primates and other mammals [14, 15, 63]. Our results suggest that this pattern holds true for *R. roxellana* as well. For instance, *Bacteroides*, a predominant genus in the human gut microbiota [51], exhibited a higher relative abundance in the gut microbiota of captive populations compared to the wild group (Figure S2).

**Fig. 6:**
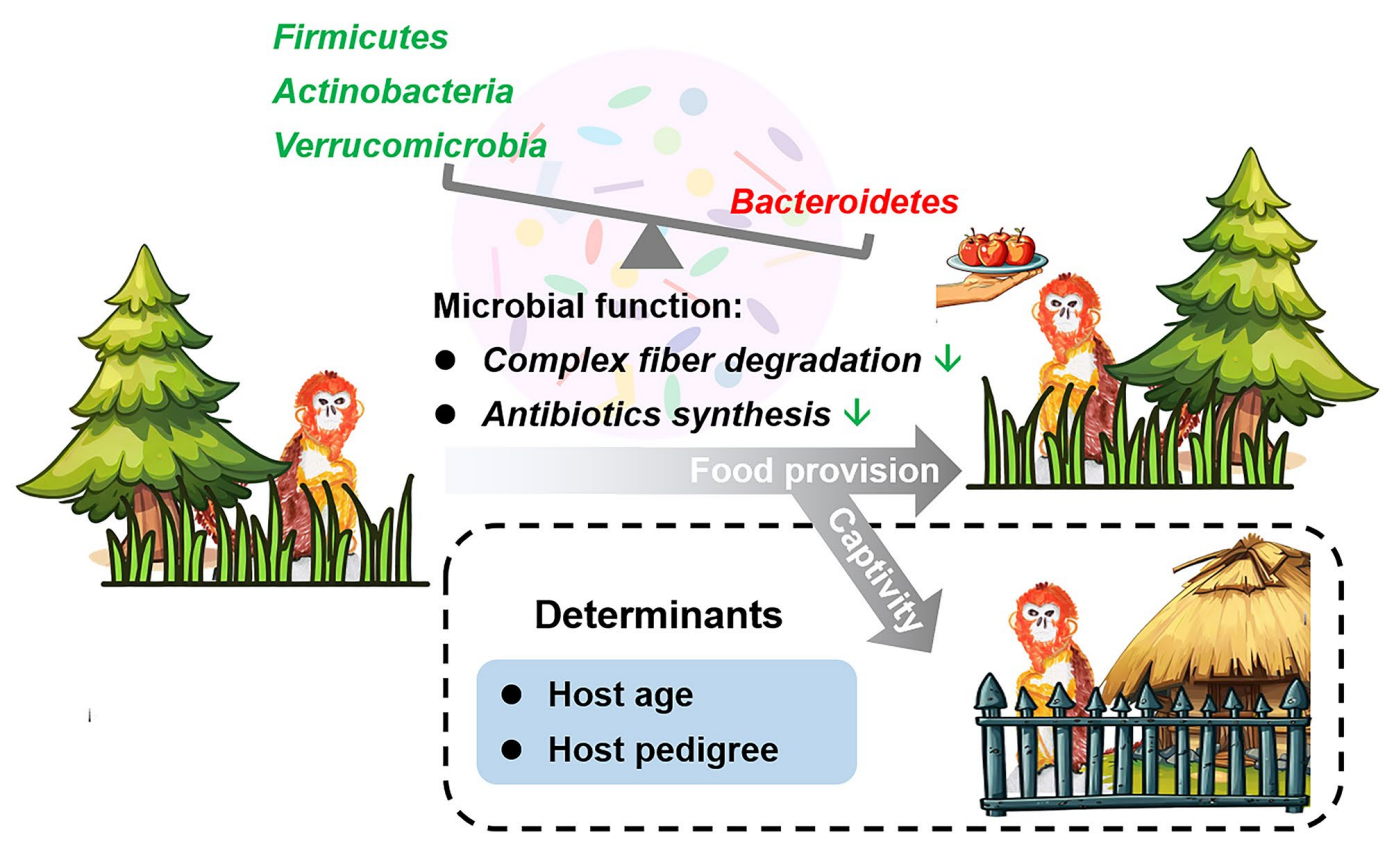
A schematic diagram summarizing the effects of anthropogenic activities and host factors on the gut microbiota of *R. roxellana*

Functional analyses indicated that AD populations exhibited a reduced capacity for antibiotics biosynthesis and the degradation of secondary compounds (Figs. 4c and 6). Wild *R. roxellana* individuals, foraging in diverse environments during different seasons, consume buds, bark, and lichen [64]. Lichen and bark are significant sources for the Actinobacteria [65–67], bacteria responsible for producing a significant portion of clinically used antibiotics [68]. In contrast, AD populations, likely foraging in less diverse environments due to artificial food provision, may experience a loss of certain microbial functions in their gut. The potential decrease in microbial antibiotics biosynthesis in captive individuals could contribute to their heightened susceptibility to gastrointestinal diseases. Remarkably, the gut microbiota of wild populations harbors a greater number of genes involved in flagellar assembly compared to AD populations (Fig. 4d − e). Bacterial antigens associated with flagella have been implicated in colitis and inflammatory bowel disease [69]. This contradicts the increased susceptibility of AD populations to gastrointestinal diseases. The “old friends hypothesis” [70] may shed light on this discrepancy. Early exposure to specific microbes is essential for developing a healthy immune system. This interaction can modulate host’s immune system, potentially diminishing immune or allergic reactions and making these microbes less likely to be recognized as pathogenic [71]. The abundant flagellar assembly genes in wild individuals may result from early and prolonged microbe-host interaction. Conversely, most captive individuals being born in captivity may lack early exposure to these bacteria, rendering their immune system more reactive to exogenous microbes and potentially inducing pathological responses. Further research is needed to validate this speculation.

Although all AD populations shared common microbial changes compared to the wild population, the SHWP and BJZ populations exhibited the most pronounced variations, particularly in the Firmicutes/Bacteroidetes ratio and microbial functional changes. In contrast, the NJZ and CDZ populations, along with the wild-fed population, showed minimal changes in their microbial traits, implying a health status similar to that of wild individuals in terms of microbial symbiosis. If the climatic factors can be excluded as significant contributors to these differences, the experiences of the NJZ and CDZ may offer valuable insights for other organizations.

### Drivers of the variations in gut microbiota of *R. roxellana*

Our findings indicate that artificial food provision can impact the gut microbiota of wild *R. roxellana*, consistent with the observation of previous studies [8, 30]. The substantial impact of diet on the gut microbiota has been well established in primates and other mammals [17, 37, 72–78]. Given that captivity inevitably leads to changes in the dietary composition of *R. roxellana*, the significant differences in the gut microbiota between captive and wild populations may also be partly explained by alterations in their dietary composition.

The gut microbiota of non-human primates may vary with host gender and age [33, 79–82]. This is consistent with our observations. The difference in microbial composition between males and females was marginally significant (Fig. 5b). Female *R. roxellana* tended to have higher microbial alpha-diversity in their gut than males (Figure S3). Unlike the gender-related variation, the age-related microbial changes in captive *R. roxellana* were much more significant (Fig. 5c − d). The variation of gut microbiota with host ages has been widely reported in primates, but the variation trends differ between species. Studies on chimpanzees and marmoset suggest that gut microbial diversity indices negatively vary with the host age [31, 38, 83], while those on *R. roxellana* and human infant indicate positive associations [81, 84]. Our results are in line with the results of prior studies on *R. roxellana*, suggesting microbial colonization in captive *R. roxellana* is an ongoing process. Age-related microbial variation in marmoset and rhesus macaques are characterized by decreased Proteobacteria and/or increased Firmicutes [31, 80, 85], while in crab-eating macaques and humans, it is associated with change in the Firmicutes/Bacteroidetes ratio [86, 87]. In this study, we did not observe significant microbial changes at the phylum level, potentially due to the large variation in the abundances of these phyla between populations. Alternatively, we observed several differential bacterial genera or OTU whose abundances increased with host age. These included an OTU belonging to Ruminococcaceae, a bacterial family whose abundance decreased in the captive individuals. These results implied that providing additional microbial sources (e.g., wild environmental microbiota) may promote the establishment of a mature gut microbiota of captive *R. roxellana*.

As anticipated, captive *R. roxellana* with closer genetic relatedness shared more similar gut microbiota (Fig. 6). However, we did not observe significant associations between maternal relatedness and the gut microbiota. These results supported the role of heredity in shaping the gut communities of primates [41]. Therefore, for captive populations, we should consider the potential effects of inbreeding on the microbial diversity and try to avoid microbial homogenization. Instead, efforts should be made to enhance genetic and behavioral interactions between different populations to improve microbial diversity.

There were two major limitations in this study. Firstly, the inclusion of only one wild population complicates the determination of whether the variances observed between wild and captive populations stem from anthropogenic disturbances or merely reflect inherent differences between populations. Secondly, the functional analyses relied on the outcomes of 16 S rRNA gene amplicon sequencing. Additional investigations, employing metagenomics, are imperative to strengthen the robustness of the conclusions.

## Conclusion

We examined the effects of anthropogenic activities and host factors on the gut microbiota of *R. roxellana* across multiple wild and captive populations. The findings underscored significant alterations in the gut microbiota induced by both captivity and artificial food provision, highlighting diet as a primary driver of these changes. The AD populations exhibited shared microbial shift, characterized by increased Bacteroidetes and decreased Firmicutes (e.g., *Ruminococcus*), Actinobacteria (e.g., *Parvibacter*), Verrucomicrobia (e.g., *Akkermansia*), and Tenericutes, along with a reduced Firmicutes/Bacteroidetes ratio. The gut microbiota of AD populations showed increased vitamin and amino acid metabolism, while decreased antibiotics biosynthesis and secondary metabolite degradation. These microbial changes might partly explain the heightened gastrointestinal susceptibility of captive individuals, with the degree of variation as potential indicators for assessing the health status of captive *R. roxellana*. For captive individuals, their gut microbial variation was driven by host age and genetic background (Fig. 6). These findings could aid in the establishment of health assessment standards and the optimization of breeding conditions for captive populations.

### Electronic supplementary material

Below is the link to the electronic supplementary material.

Supplementary Material 1

Supplementary Material 2

## Data Availability

Sequencing data and relevant files have been uploaded to Genome Sequence Archive (https://ngdc.cncb.ac.cn/gsub/) with the accession number CRA011956 (https://ngdc.cncb.ac.cn/gsa/s/y0jKJ234).
