# Supplementary material for: Comparing the gut microbiota of Sichuan golden monkeys across multiple captive and wild settings: roles of anthropogenic activities and host factors
Source: BMC Genomics. 2024 Feb 6;25:148. doi: 10.1186/s12864-024-10041-7 (PMC10848473; doi:10.1186/s12864-024-10041-7)
Supplement: Supplementary file 2 — Supplementary Material 2 [file 12864_2024_10041_MOESM2_ESM.docx]

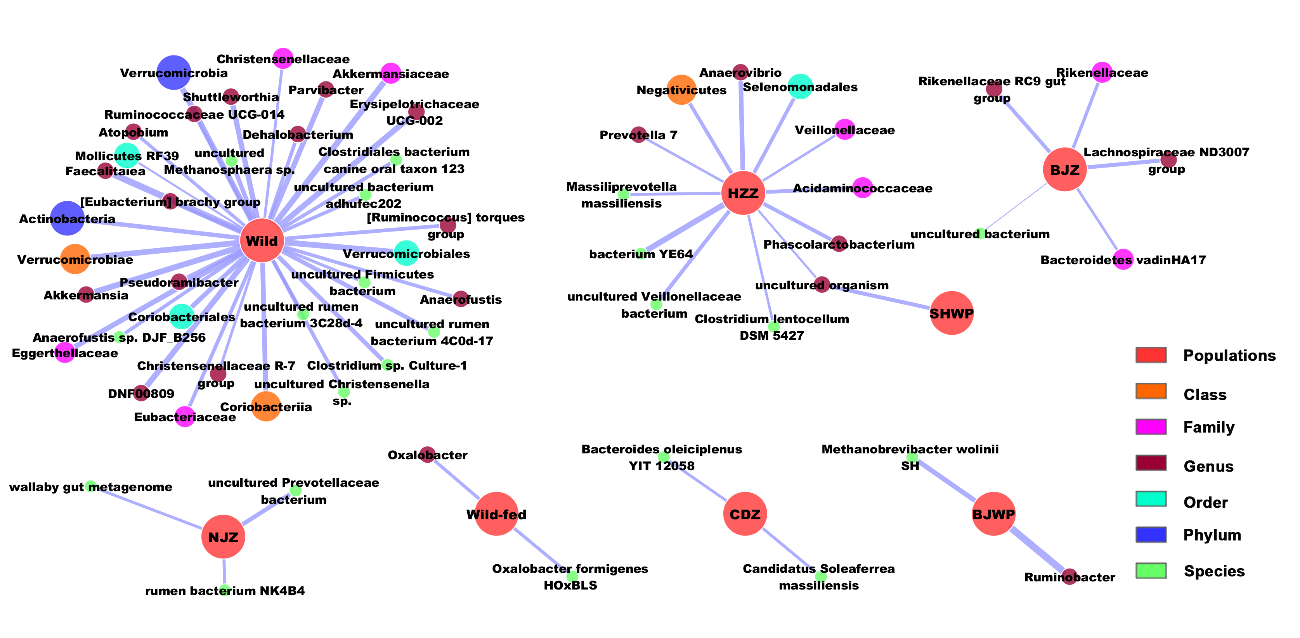


**Figure S1** The indicative bacterial taxa of each animal population. The node size denotes the relative abundance of the bacterial taxa, and the edge width indicates the correlation coefficient.





**Figure S2** Comparison of the relative abundance of *Bacteroides* in gut microbiota between *R. roxellana* and humans. Captivity likely humanizes the gut microbiota of *R. roxellana*. The human data were from a study conducted on volunteer from George Washington University (GW) Foggy Bottom campus area [[1](#_ENREF_1)].


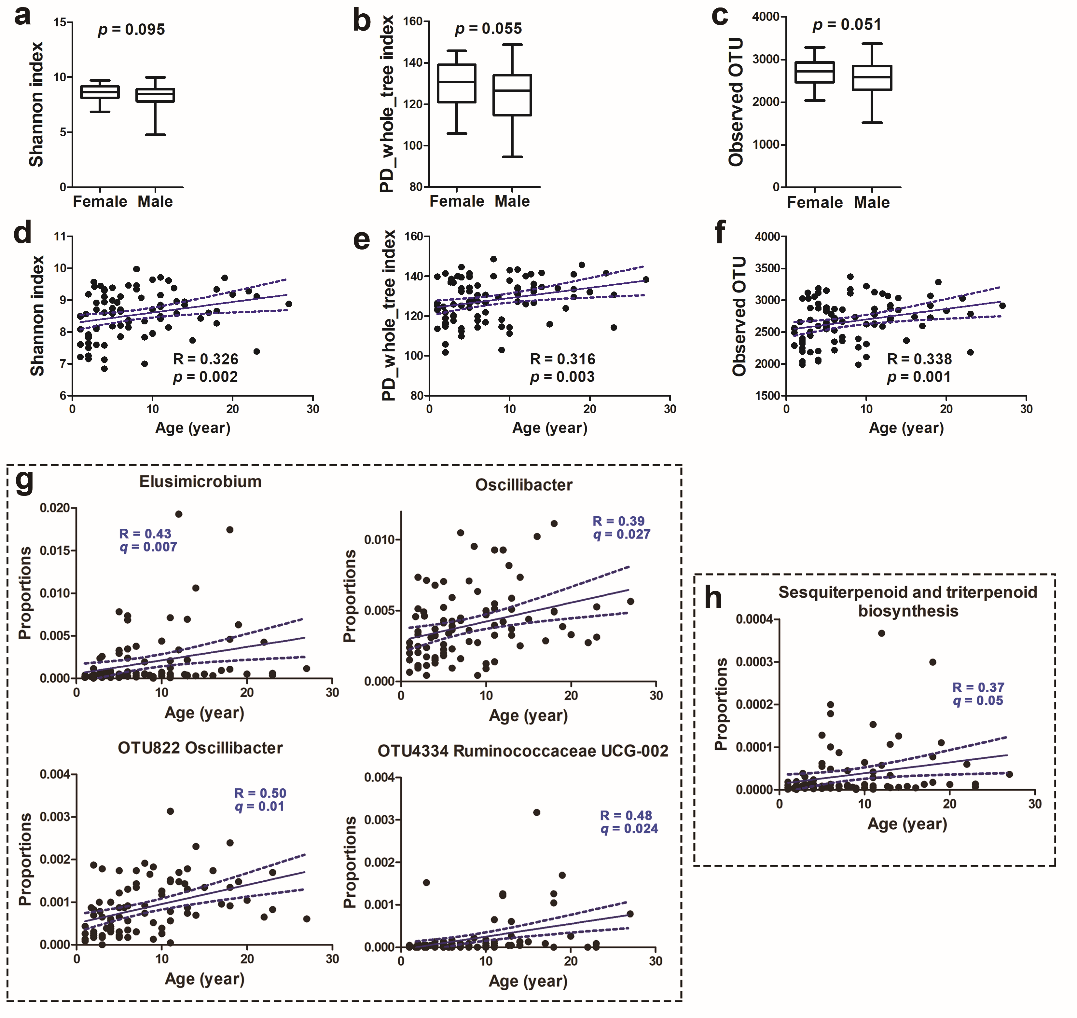


**Figure S3** Influences of host factor on the gut microbiota. (a−c) Influences of host gender on the bacterial alpha-diversity (Mann-Whitney U tests). (d−f) Influences of host age on the bacterial alpha-diversity (Spearman correlation). (g−h) Microbial taxa (c) and functions (d) that varied significantly with host age (Spearman correlation).

1. King CH, Desai H, Sylvetsky AC, LoTempio J, Ayanyan S, Carrie J, Crandall KA, Fochtman BC, Gasparyan L, Gulzar N *et al*: **Baseline human gut microbiota profile in healthy people and standard reporting template**. *PloS one* 2019, **14**(9):e0206484.
